# Smoking-Induced DNA Hydroxymethylation Signature Is Less Pronounced than True DNA Methylation: The Population-Based KORA Fit Cohort

**DOI:** 10.3390/biom14060662

**Published:** 2024-06-05

**Authors:** Liye Lai, Pamela R. Matías-García, Anja Kretschmer, Christian Gieger, Rory Wilson, Jakob Linseisen, Annette Peters, Melanie Waldenberger

**Affiliations:** 1 Research Unit of Molecular Epidemiology, Helmholtz Zentrum München, German Research Center for Environmental Health (GmbH), 85764 Neuherberg, Germany; pamela.matiasgarcia@helmholtz-munich.de (P.R.M.-G.); christian.gieger@helmholtz-munich.de (C.G.); wilson.rory@gmail.com (R.W.); annette.peters@helmholtz-munich.de (A.P.); 2 Institute for Medical Information Processing, Biometry, and Epidemiology (IBE), Pettenkofer School of Public Health, Faculty of Medicine, Ludwig Maximilians University, 81377 Munich, Germany; 3 Institute of Epidemiology, Helmholtz Zentrum München, German Research Center for Environmental Health (GmbH), 85764 Neuherberg, Germany; anja.kretschmer@helmholtz-munich.de; 4 Epidemiology, Faculty of Medicine, University Hospital of Augsburg, University of Augsburg, 86156 Augsburg, Germany; jakob.linseisen@med.uni-augsburg.de; 5 German Centre for Cardiovascular Research (DZHK), Partner Site Munich Heart Alliance, 81377 Munich, Germany

**Keywords:** smoking, DNA methylation, hydroxymethylation, differentially methylated positions (DMPs), differentially methylated regions (DMRs), Illumina Infinium Methylation EPIC BeadChip

## Abstract

Despite extensive research on 5-methylcytosine (5mC) in relation to smoking, there has been limited exploration into the interaction between smoking and 5-hydroxymethylcytosine (5hmC). In this study, total DNA methylation (5mC+5hmC), true DNA methylation (5mC) and hydroxymethylation (5hmC) levels were profiled utilizing conventional bisulphite (BS) and oxidative bisulphite (oxBS) treatment, measured with the Illumina Infinium Methylation EPIC BeadChip. An epigenome-wide association study (EWAS) of 5mC+5hmC methylation revealed a total of 38,575 differentially methylated positions (DMPs) and 2023 differentially methylated regions (DMRs) associated with current smoking, along with 82 DMPs and 76 DMRs associated with former smoking (FDR-adjusted *p* < 0.05). Additionally, a focused examination of 5mC identified 33 DMPs linked to current smoking and 1 DMP associated with former smoking (FDR-adjusted *p* < 0.05). In the 5hmC category, eight DMPs related to current smoking and two DMPs tied to former smoking were identified, each meeting a suggestive threshold (*p* < 1 × 10^−5^). The substantial number of recognized DMPs, including 5mC+5hmC (7069/38,575, 2/82), 5mC (0/33, 1/1), and 5hmC (2/8, 0/2), have not been previously reported. Our findings corroborated previously established methylation positions and revealed novel candidates linked to tobacco smoking. Moreover, the identification of hydroxymethylated CpG sites with suggestive links provides avenues for future research.

## 1. Introduction

Although tobacco smoking is widely recognized as a harmful behaviour with significant impacts on human health, smoking or exposure to smoke continues to be prevalent worldwide. Tobacco smoking is a risk factor for and is a frequent cause of many adverse health consequences, such as chronic obstructive pulmonary disease (COPD) [1], cardiovascular diseases [2], asthma [3] and various forms of cancer, in particular lung cancer [4,5]. Moreover, smoking status appears to contribute to a poor prognosis in COVID-19 patients [6]. While the precise pathogenic mechanisms remain under investigation, it is widely acknowledged that the induction of oxidative stress through the generation of excessive reactive oxygen species (ROS) by harmful chemicals is a key molecular event that predisposes individuals to inflammation, senescence and smoking-related illnesses [7,8].

Epigenetic mechanisms, specifically alterations in DNA methylation, have been suggested to moderate the impact of tobacco smoking, leading to changes in transcriptional activity and contributing to smoking-related diseases [9]. With the update of DNA methylation arrays, the impact of smoking on DNA 5-methylcytosine (5mC) methylation has been thoroughly investigated in blood cells from adults, revealing significant disparities between smokers and non-smokers [10,11], which can be even more conspicuous in specific tissues like vascular endothelial cells [12], and vulnerable groups like cancer patients [4]. The impact of tobacco smoking on DNA methylation is also prominent in the blood of newborns whose mothers smoked during pregnancy [13]. Previous studies also demonstrated that the link between cigarette smoking and methylation is dynamic, showing ongoing fluctuations in methylation levels even decades after smoking cessation. However, only a few studies have delved into the effect of smoking on DNA 5-hydroxymethylcytosine (5hmC) methylation, an intermediate oxidized form of 5mC involved in the active demethylation process. During active demethylation process, the ten-eleven translocation (TET) enzymes play a crucial role by oxidizing 5mC into 5hmC, further converting 5hmC to 5-formylcytosine (5fC) and 5-carboxylcytosine (5caC). Subsequently, the thymine DNA glycosylase (TDG)-dependent base excision repair (BER) transforms 5fC and 5caC into an unmethylated cytosine [14,15]. Due to their low abundance in the genome, 5fC and 5caC demonstrate limited stability [16]. In contrast to 5fC and 5caC, 5hmC is relatively stable and presents tissue specificity [17]. Given its enrichment in promoters, enhancers and transcriptional regulatory elements, 5hmC is intimately associated with the regulation of gene expression [18].

**Figure 1:**
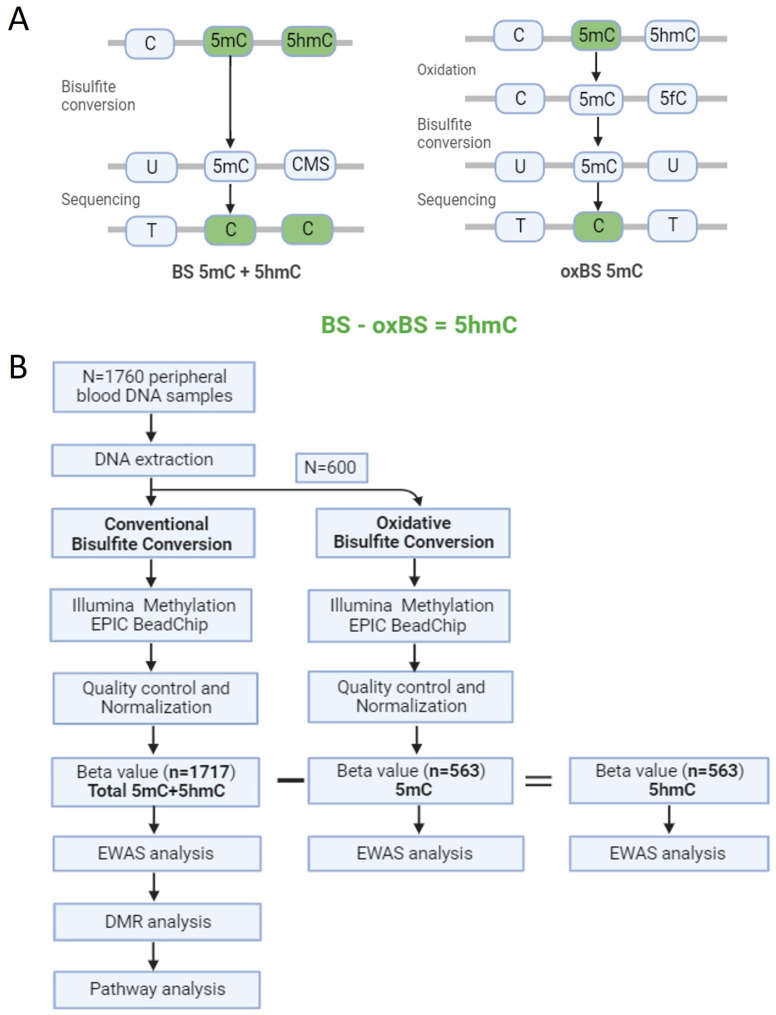
(**A**) Schematic overview depicting bisulphite conversion (BS) and oxidative BS. (**B**) Illustration of the study design.

Recent studies have highlighted that smoking-induced oxidative stress can initiate the DNA demethylation pathway [19]. Additionally, 5hmC has emerged as an informative biomarker in mammalian development and diseases [20,21]. However, the traditional bisulphite (BS) conversion method, commonly used for detecting DNA methylation, cannot distinguish between 5mC and 5hmC [22]. As a result, most of the existing literature on DNA methylation reports 5mC and 5hmC signals jointly. Moreover, the Infinium HumanMethylation450 BeadChip has been predominantly utilized to identify smoking-associated differentially methylated positions (DMPs). In this study, the oxidative bisulphite (oxBS) treatment was employed to measure true 5mC and 5hmC signals separately (Figure 1A). We hypothesized that smoking-induced differential DNA methylation could potentially influence not only 5mC but also 5hmC patterns in leucocytes from blood samples. Initially, we examined total 5mC+5hmC methylation levels in 1717 participants classified as current, former and non-smokers from the Cooperative Health Research in the Region of Augsburg (KORA) Fit population-based cohort (Figure 1B). We employed the latest HumanMethylation EPIC BeadChip, providing expanded CpG site coverage compared to prior arrays (over 850,000 CpG sites). Subsequently, we evaluated 5mC and 5hmC methylation levels separately in a subset of 563 individuals.

## 2. Materials and Methods

### 2.1. Study Population

The analysis was based on data from the KORA Fit study, a follow-up study conducted between early 2018 and mid-2019, building upon the 4 cross-sectional baseline surveys (KORA S1, S2, S3 and S4 cohorts). All living participants of the KORA cohorts born between 1945 and 1964 who consented to be recontacted were invited for a new examination (*n* = 3059 or 64.4% of all eligible participants). Exhaustive information about this study has been described previously [23]. In total, 1760 participants with available data on DNA methylation were included in the analysis. Specifically, for the investigation into true methylation and hydroxymethylation, a subgroup comprising 600 participants from the KORA Fit study was considered. This subgroup included individuals who participated in both the S4 baseline survey and the KORA Fit examination. Individuals who self-declared as either regular or occasional smokers (defined as 1 cigarette per day or less) at the time of the interview were classified as current smokers. Those who had never smoked were categorized as non-smokers, while individuals who had previously smoked but were not currently smoking at the time of the interview were classified as former smokers.

### 2.2. DNA Extraction and DNA Methylation Quantification

DNA extraction followed standard procedures. For the total 5mC+5hmC methylation processing, genomic DNA (750 ng) from 1160 individuals underwent BS conversion using the EZ-96 DNA Methylation Kit (Zymo Research, Orange, CA, USA). Meanwhile, genomic DNA (1500 ng) from 600 individuals was split (750 ng each), and separate aliquots of each DNA sample were processed in parallel. One aliquot underwent BS treatment to generate total methylation (5mC+5hmC) signals, while the other aliquot underwent oxidation and then BS treatment to generate true methylation (5mC) signals, both using the TrueMethyl oxBS Module (Tecan Genomics, Redwood City, CA, USA). During BS treatment, 5mC and 5hmC are preserved as cytosines, whereas unmethylated cytosines are deaminated to uracil. Consequently, DNA methylation measured by the BS treatment reflects an amalgamation of 5mC and 5hmC. Upon oxidation, 5mC remains as 5mC, while 5hmC is converted into 5fC. The 5fC is susceptible to BS treatment, and it is deaminated into uracil (equivalent to an unmethylated cytosine), while 5mC is preserved as a cytosine upon BS treatment. Thus, oxBS conversion enables the specific measurement of nucleotide-level 5mC [24,25]. Subsequent methylation analysis for all samples was conducted on an Illumina (San Diego, CA, USA) iScan platform using the Infinium Methylation EPIC BeadChip v1, following standard protocols provided by Illumina. Initial quality control procedures of assay performance and generation of methylation data export files were carried out using GenomeStudio software version 2011.1 with Methylation Module version 1.9.0.

### 2.3. Preprocessing and Normalization

Raw intensities were imported, and further quality control and preprocessing were performed in R software (R v4.3.3), with the minfi package v1.48.0, primarily following the CPACOR pipeline [26]. Total methylation (5mC+5hmC) and true methylation (5mC) were processed separately. Samples with defective chips and over 20% missing values, along with sex-mismatching samples, were removed. Probes with detection *p*-values great than 0.01 in more than 5% of samples were set to missing. Furthermore, sex chromosomes and cross-reactive and SNP-related probes were removed. Subsequently, quantile normalization (QN) was independently performed on the signal intensities, which were categorized into the 6 probe types: type II red, type II green, type I green unmethylated, type I green methylated, type I red unmethylated, type I red methylated. β-values were then calculated by initiating with the BS signal, representing the total methylation (5mC+5hmC) signal at each CpG site. Total methylation β-values were computed as the ratio of the methylated signal over the sum of the methylated and unmethylated signals [27]. For the analysis of total 5mC+5hmC methylation, 1717 samples and 734,349 probes were retained for the final analysis. Similarly, 5mC β-values were calculated using the oxBS signal. Lastly, the level of 5hmC at a single-nucleotide resolution was estimated by subtracting the oxBS measure (5mC) from the BS measure (5mC+5hmC) at each probe. Specifically, for the hydroxymethylation, only probes and samples that were common between the 5mC+5hmC and 5mC datasets were kept, resulting in 563 samples and 756,737 probes. Additionally, subtracting 5mC from 5mC+5hmC is known to introduce negative β-values, so any negative β-values were set to a value close to zero (1 × 10^−7^).

### 2.4. Differential Methylation Analysis

An Epigenome-wide association study (EWAS) was carried out using a multivariate linear regression model, where smoking status (current, former, non-smokers) served as the exposure variable, and untransformed methylation β-values (ranging from 0 to 1) were used as the outcome. Recognizing that methylation levels in blood can be significantly influenced by leukocyte composition, the houseman algorithm was employed to estimate white blood cell type proportions [28]. Additionally, principal components (PCs) of all non-negative control probes were calculated to account for technical effects. All epigenome-wide analyses were adjusted for the age at blood collection, sex, BMI, six estimated cell type proportions (monocytes, granulocytes, natural killer cells, B cells, CD4T cells and CD8T cells) and the first 5 principal components (PCs). To assess the epigenome-wide distribution of *p* values compared to the expected null distribution of *p* values, we calculated the inflation factor λ and generated quantile–quantile (QQ) plots. The inflation factor was defined as the ratio of the median of the observed log10-transformed *p* values to the median of the expected log10-transformed *p* values. We also applied bacon correction to mitigate bias and inflation of the test statistic. A probe was considered significantly differentially methylated with a false discovery rate (FDR)-adjusted (Benjamini–Hochberg) *p* value less than 0.05. Given the anticipated lower range of 5hmC methylation values, a less stringent suggestive threshold of *p* < 1 × 10^−5^ was employed when identifying 5hmC-associated differential methylation. EWAS Catalog (a database of epigenome-wide association studies) [29] was used to compare and select the novel smoking-associated CpG candidates. DMRs represent genomic regions with consistently different DNA methylations across multiple adjacent CpG sites. In addition to the single-site DMP analysis, we applied the comb-p function using the Enmix package (version 1.38.01), which provides quality control, analysis and visualization tools for Illumina DNA methylation BeadChip, to detect DMRs among current, former and non-smokers. In this analysis, regions were defined as sets of all probes containing ≥3 DMPs within 1000 base pairs of another probe and having false discovery rate (FDR)-adjusted *p* values less than 0.05.

### 2.5. Gene Enrichment Analyses

To gain insights into potential smoking-relevant biological processes, gene pathway analysis was performed in the context of differentially methylated CpG sites. This analysis utilized the GOmeth function from the missMethyl package (version 1.38.0), which accounts for the number of CpG sites per gene on the 450K/EPIC array and multi-gene-annotated CpGs. Independent pathways with an FDR *p* < 0.05 were considered significantly associated with smoking. Gene annotation was performed using the HumanMethylation EPIC probe annotation file.

## 3. Results

### 3.1. Characteristics of the Study Population

A total of 1717 participants were included in our study for further analyses after quality control, consisting of 217 current smokers, 719 former smokers and 781 non-smokers. The cohort characteristics are described in Table 1. Current smokers were younger and exhibited a lower prevalence of hypertension compared to non-smokers. Former smokers had a larger proportion of males and a higher BMI level. Both current and former smokers displayed an increased daily alcohol consumption, lower HDL cholesterol levels and higher triglycerides levels. All groups were comparable in terms of physical activity, diabetes status, HOMA-IR and HOMA-Beta levels.

### 3.2. Distribution of Methylation β-Values

**Figure 2:**
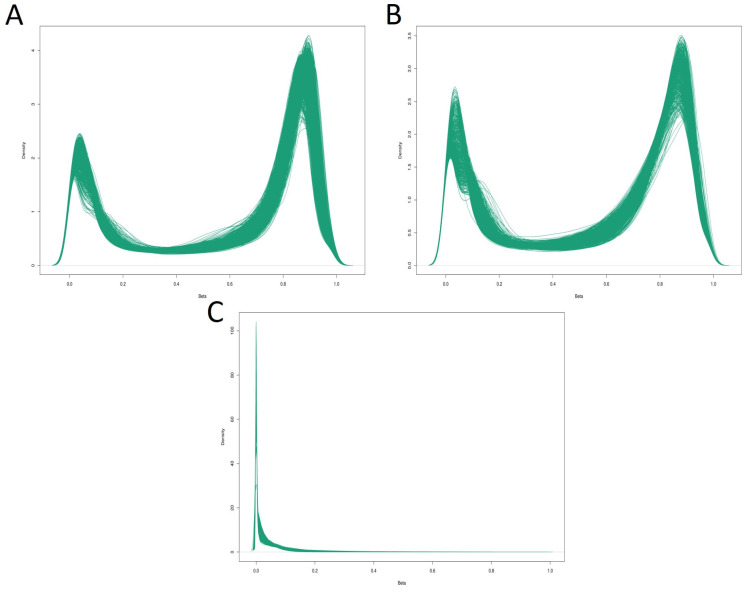
Density plots illustrating the distribution of methylation β-values. The *x*-axis represents the β-values ranging from 0 to 1, while the *y*-axis depicts the corresponding density. (**A**) Density plot for total 5mC+5hmC methylation β-values. (**B**) Density plot for true 5mC methylation β-values. (**C**) Density plot for 5hmC hydroxymethylation β-values.

The methylation β-values, ranging from 0 to 1, were computed as the ratio of the methylated signal to the sum of the methylated and unmethylated signals. The distribution of methylation β-values are described in Figure 2. The distribution of β-values for total 5mC+5hmC and 5mC methylation were notably similar, with the median values of 0.75 (interquartile range (IQR) = 0.03) and 0.56 (IQR = 0.03), respectively. Both distributions follow an obvious binomial pattern, drastically compressed within the low (0–0.2) and high (0.8–1.0) ranges. However, the values for 5hmC were notably low, with a median value of 0.03 (IQR = 0.02).

### 3.3. Site-Specific Changes in Total 5mC+5hmC Associated with Smoking

The EWAS was conducted to determine epigenome-wide differences in total 5mC+5hmC methylation among current, former and non-smokers. Additionally, we employed bacon correction to mitigate bias and inflation of the test statistic, resulting in a correction of the inflation factor to 1.38 (Supplementary Material S1: Figure S1A,B), which is consistent with many CpG sites being impacted by tobacco smoking. The analysis of 5mC+5hmC methylation data revealed 38,575 DMPs associated with current smoking and 82 DMPs associated with former smoking (FDR-adjusted *p* < 0.05). A summary of the top 10 most significant 5mC+5hmC DMPs associated with both current and former smoking is shown in Table 2, and the complete list of significant 5mC+5hmC DMPs can be found in Supplementary Material S2: Tables S1 and S2.

**Figure 3:**
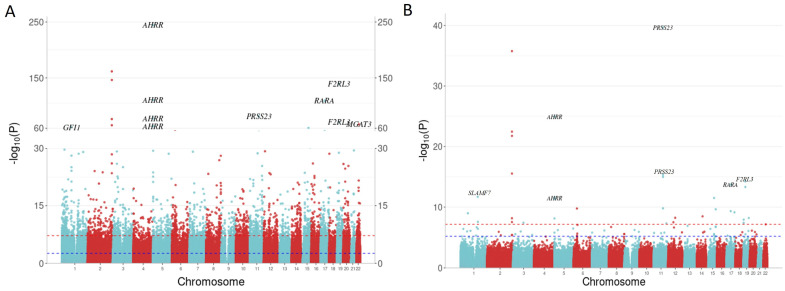
Manhattan plots illustrating smoking EWAS results for 5mC+5hmC methylation. The *x*-axis indicates the chromosome location, and the *y*-axis represents the −log10 (*p*-value). The Bonferroni threshold of 6.81 × 10^−8^ is marked by a red dashed line, while the Benjamini–Hochberg (FDR) threshold (*p* < 0.05) is indicated by a blue dashed line. The ggbreak package (version 0.1.2) was used to effectively utilize plotting space and handle large *y*-axis values for currents smokers. (**A**) Manhattan plot for current vs. non-smokers; (**B**) Manhattan plot for former vs. non-smokers.

The results supported many previously reported gene loci, including CpG sites annotated to aryl hydrocarbon receptor repressor (*AHRR*), retinoic acid receptor alpha (*RARA*), F2R-like thrombin or trypsin receptor 3 (*F2RL3*) and serine protease 23 (*PRSS23*). Notably, cg05575921 (annotated to *AHRR*), which has consistently emerged as the most significant DMP in previous smoking studies, demonstrated remarkable significance (*p* = 1.56 × 10^−239^) and exhibited the largest effect size in our analysis (−22.72% difference in methylation). Out of the 38,575 DMPs, 59.32% (22,884/38,575) were exclusive to EPIC BeadChip and did not present on the previous 450k BeadChip. Moreover, 18.33% (7069/38,575) of the DMPs were novel candidates, not previously reported in the EWAS Catalog (Supplementary Material S2: Table S3). A predominant fraction of DMPs, comprising 77.71% (29,977/38,575), exhibited hypomethylation due to current smoking, with a mean methylation difference of 1.07% (SD = 0.53%). Conversely, 22.29% (8598/38,575) of the DMPs displayed hypermethylation, showing a mean percentage difference of 1.03% (SD = 0.53%). The Manhattan plot (Figure 3A) and the Volcano plot (Supplementary Material S1: Figure S2A) illustrated EWAS results for 5mC+5hmC methylation related to current smoking.

In former smokers, only 82 CpG sites remained differentially methylated, although with reduced effect sizes compared to the observed effects in current smokers. Genomic inflation was not strongly evident (λ = 1.13). All annotated genes associated with former smoking, including *PRSS23*, *AHRR*, *F2RL3* and *RARA*, overlapped with genes associated with current smoking. In contrast to current smokers, the most significant CpG site in former smokers was cg14391737, annotated to *PRSS23* (*p* = 1.63 × 10^−34^, effect size: −4.56%), surpassing cg05575921, annotated to *AHRR* (*p* = 2.95 × 10^−20^, effect size: −4.06%). Of the 82 identified DMPs, 51.22% (42/82) were exclusive to the EPIC BeadChip and 2.44% (2/82) DMPs were novel candidates (Supplementary Material S2: Table S4). For 90.24% (74/82) of DMPs displaying decreased methylation in response to former smoking, the mean methylation percentage difference was 1.37% (SD = 0.78%). For 9.76% (8/82) of DMPs showing increased methylation in response to former smoking, the mean percentage difference was 1.55% (SD = 0.67%). The Manhattan plot (Figure 3B) and the Volcano plot (Supplementary Material S1: Figure S2B) illustrate EWAS results for 5mC+5hmC methylation related to former smoking.

### 3.4. Site-Specific True Methylation Changes Associated with Smoking

**Figure 4:**
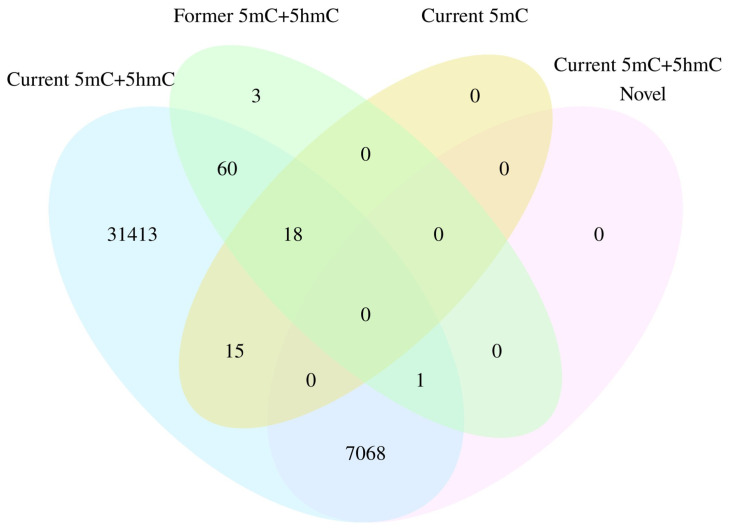
Venn plot illustrating the overlap number of DMPs in different methylation dataset. The blue and cyan colours represent the number of significant DMPs, related with current and former smoking respectively, in the context of 5mC+5hmC methylation. The yellow colour represents the number of significant DMPs related with current smoking in the context of 5mC methylation. The pink colour represents the number of novel DMPs related with current in the context of 5mC+5hmC methylation.

**Figure 5:**
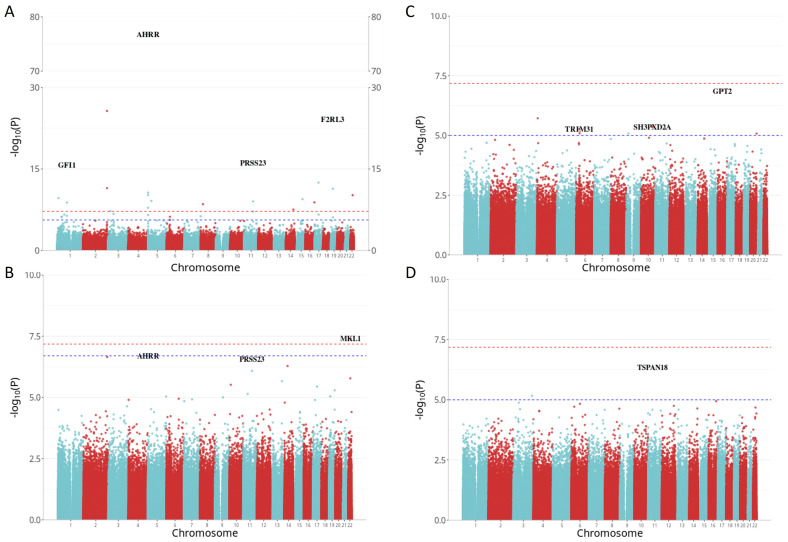
Manhattan plots illustrating smoking EWAS results for both 5mC and 5hmC methylation. The *x*-axis represents the chromosome location, while the *y*-axis represents the −log10(*p* value). The Bonferroni threshold of 6.61 × 10^−8^ is marked by a red dashed line, while the Benjamini–Hochberg (FDR) threshold (*p* < 0.05) is indicated by a blue dashed line. The ggbreak packagewas used to effectively utilize plotting space and handle large *y*-axis values for currents smokers. (**A**) Manhattan plot for current vs. non-smokers in 5mC dataset; (**B**) Manhattan plot for former vs. non-smokers in 5mC dataset; (**C**) Manhattan plot for current vs. non-smokers in 5hmC dataset; (**D**) Manhattan plot for former vs. non-smokers in 5hmC dataset.

True DNA methylation (5mC) was measured by oxBS treatment. A total of 33 DMPs were associated with current smoking and 1 5mC DMP was identified between former vs. non-smokers. There was no evidence of inflation (λ = 0.996 for current smokers, λ = 1.009 for former smokers). The count of 5mC DMPs for both current and former smoking was prominently lower than of 5mC+5hmC DMPs. Remarkably, all 33 of the 5mC DMPs, linked to current smoking, were encompassed within the 5mC+5hmC results (Figure 4), and the overall pattern of the 5mC+5hmC and 5mC methylation changes exhibited similarity. For example, the cg05575921, annotated to *AHRR*, consistently retained its position as the most strongly associated with current smoking (*p* = 1.27 × 10^−77^) and showed a slightly stronger effect size difference (−24.01%) in the 5mC methylation dataset. In line with 5mC+5hmC, 72.73% (24/33) of the DMPs exhibited hypomethylation in the 5mC dataset, demonstrating a mean difference in methylation of −7.75% (SD = 4.46%). Additionally, 27.27% (9/33) of the DMPs displayed hypermethylation with a mean difference in methylation of −7.09% (SD = 1.66%). For former smokers, only cg24476099, annotated to megakaryoblastic leukemia 1 (*MKL1*), reached statistical significance with an effect size of −4.34%, and it is specific to the EPIC BeadChip. The most significant 5mC DMPs are shown in Table 3, and the complete list can be found in Supplementary Material S2: Tables S5 and S6. The Manhattan plot (Figure 5A,B) and Volcano plot (Supplementary Material S1: Figure S4A,B) illustrate EWAS results for 5mC methylation related to current and former smoking.

### 3.5. Site-Specific Hydroxymethylation Changes Associated with Smoking

The total 5mC+5hmC methylation levels were determined using BS treatment, while true DNA methylation (5mC) was measured by oxBS treatment. The quantification of 5hmC involved subtracting 5mC β-values from the combined 5mC+5hmC β-values. 5hmC methylation values were observed at a lower level, so a suggestive threshold of *p* < 1 × 10^−5^ was set, revealing eight and two significant 5hmC DMPs between current vs. non-smokers and former vs. non-smokers, respectively. No strong evidence of inflation was detected (λ = 1.132 for current smokers, λ = 1.018 for former smokers). The cg16972043, annotated to the glutamate pyruvate transaminase 2 (*GPT2*) gene, emerged as the most strongly associated (*p* = 1.26 × 10^−7^) with current smoking and displayed the largest effect size difference (4.14%) in the 5hmC methylation dataset. Conversely, the cg24012880, annotated to the tetraspanin 18 (*TSPAN18*) gene, demonstrated the strongest association (*p* = 4.45 × 10^−7^) with former smoking, displaying an effect size difference of 3.61%. In contrast with methylation changes observed in 5mC+5hmC and 5mC datasets, almost all the top 5hmC DMPs were hypermethylated, demonstrating a mean methylation difference of 2.32% (SD = 1.11%) in current smokers and 0.99% (SD = 0.04%) in former smokers. The most significant 5hmC DMPs are shown in Table 3, and the complete list can be found in Supplementary Material S2: Tables S7 and S8. The Manhattan plot (Figure 5C,D) and the Volcano plot (Supplementary Material S1: Figure S4C,D) illustrated EWAS results for 5hmC methylation associated with current and former smoking.

### 3.6. Region-Specific Changes Associated with Smoking

In the total 5mC+5hmC dataset, there were 2023 distinct DMRs linked to current smoking, encompassing 9367 measured CpG sites annotated across 1553 genes. The most prominent DMR uncovered in individuals who currently smoke was situated in a region on chromosome 1, annotated to the growth factor independent 1 transcriptional repressor (*GFI1*) gene, spanning nine CpG sites. The DMR displaying the second strongest association comprised seven CpG sites and was annotated to *AHRR*. A substantial overlap of genes (1542/1553, 99.29%) was observed between the genes identified in the DMP and DMR analyses, which included notable genes like *GFI1*, *AHRR* and HIVEP Zinc Finger 3 (*HIVEP3*). Notably, DMR analyses produced 11 additional genes not identified in DMP analyses, such as Retinoic Acid Receptor Responder 2 (*RARRES2*), Ring Finger Protein 40 (*RNF40*) and Solute Carrier Family 1 Member 5 (*SLC1A5*). During the DMR analysis comparing former smokers and non-smokers, a total of 76 distinct DMRs were identified, containing 390 measured CpG sites and annotated to 61 different genes. Only a minimal overlap of 9.83% (6/61) was observed with previously identified DMPs, specifically Alanyl Aminopeptidase Membrane (*ANPEP*) and *PRSS23*. Additionally, 55 annotated genes such as Proline Rich Transmembrane Protein 1 (*PRRT1*) were exclusively detected in the DMR results. In the true 5mC dataset, there were 14 distinct DMRs linked to current smoking, encompassing 85 measured CpG sites annotated across 12 genes such as *HIVEP3*, *GFI1* and Valyl-TRNA Synthetase 1 (*VARS*). Additionally, there were five distinct DMRs linked to former smoking, encompassing 25 CpG sites annotated across four genes. In the 5hmC dataset, we did not find any DMRs related to current or former smoking. The top 10 most significant DMRs linked to both current and former smoking are presented in Table 4. The complete list of DMRs can be found in Supplementary Material S2: Tables S9–S12; Manhattan plots illustrating DMR results for the 5mC+5hmC and true 5mC methylation datasets related to current and former smoking can be found in Supplementary Materials S1: Figures S3 and S6.

### 3.7. Gene Enrichment Analysis

**Figure 6:**
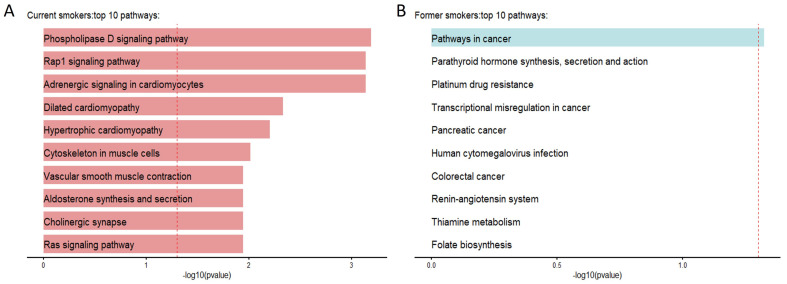
Enrichment analysis results of total 5mC+5hmC methylation. The *x*-axis represents the −log10(*p*-value), and the red dashed line represents the significant threshold (FDR-adjusted *p* < 0.05). (**A**) The top 10 most significant pathways derived from 5mC+5hmC methylation between current and non-smokers. (**B**) The top 10 most significant pathways derived from 5mC+5hmC methylation between former and non-smokers.

The genes associated with DMPs that passed the significant threshold (FDR-adjusted *p* < 0.05) were identified. Exploratory downstream enrichment analyses were performed on those genes using the missMethyl package with the KEGG dataset. In the total 5mC+5hmC methylation dataset, DMPs associated with current smoking exhibited enrichment in 27 pathways, whereas DMPs associated with former smoking showed enrichment in 1 pathway. However, we did not find any significant pathway from the true 5mC and 5hmC datasets. These findings suggest a potential link between cigarette smoking and alterations in various molecular pathways, including mechanisms of cardiovascular diseases and cancers. The top 10 ranked biological pathways based on DMPs related to current and former smoking from total 5mC+5hmC are illustrated in Figure 6. The complete lists of pathways, from the total 5mC+5hmC, true 5mC and 5hmC methylation datasets, can be found in Supplementary Material S2: Tables S13–S18.

## 4. Discussion

We have investigated different DNA methylation modifications among individuals categorized as current, former and non-smokers. This is, to the best of our knowledge, the first epigenome-wide methylation study of smoking’s effects on blood leucocyte samples, analysing true 5mC and 5hmC as distinct DNA methylation modifications, especially in conjunction with the Illumina EPIC BeadChip. Initially, we explored the association between smoking status and total 5mC+5hmC methylation levels, identifying 38,575 and 82 DMPs associated with current and former smoking, many of which are novel candidates. Subsequently, employing tandem BS and oxBS treatment, we differentiated 5hmC from 5mC at the single-nucleotide level. Within this refined analysis, we discovered 33 and 1 DMPs associated with current and former smoking in the 5mC category, respectively. Additionally, eight and two DMPs linked to current and former smoking were identified in the 5hmC category, respectively. We observed a high concordance in the direction of effects and a large overlap in the identified loci between 5mC+5hmC and 5mC groups.

Robust associations have been established between smoking exposure and alterations in blood DNA methylation, supported by the identification of numerous specific loci [11,30]. For example, the most extensive meta-analysis of smoking-associated epigenome-wide DNA methylation was conducted using the 450K array to analyse 15,907 blood-derived DNA samples from individuals across 16 cohorts. A total of 2623 CpG sites, annotated to 1405 genes, demonstrated associations with current smoking [10]. In this study, we replicated many previously reported sites, including those annotated to *AHRR*, *RARA*, *F2RL3*, *PRSS23* and *GFI1* [31], and identified a substantial number of the novel smoking-associated candidates by using the latest EPIC BeadChip. The *AHRR* gene consistently appeared as the most significantly affected genomic locus in studies investigating the impact of smoking [32,33], a pattern also evident in our cohort. Specifically, 41 DMPs associated with current smoking were annotated to *AHRR* in the 5mC+5hmC dataset, and 11 in the 5mC dataset. All these findings substantiate the robustness and reliability of our study results.

The global initiatives for smoking cessation, coupled with legislative measures, have led to a decline in the number of cigarette smokers and a concomitant rise in the population of former smokers. Decades after cessation, cigarette smoking continues to pose a long-term risk for diseases, and DNA methylation also leaves a persistent signature after smoking exposure [34]. In our analysis, despite the majority of differently methylated CpG sites returning to the methylation levels like non-smokers following smoking cessation, a subset of CpG sites exhibited sustained different methylation even after quitting smoking, albeit with diminished effect sizes in former smokers. The impact of smoking on these specific CpG sites holds the potential to function as robust biomarkers, offering insights into an individual’s historical smoking behaviour and reflecting enduring health consequences [35,36].

Clusters of neighbouring probes associated with a phenotype, known as DMRs, may enhance the ability to detect associations between DNA methylation and diseases or phenotypes of interest [37]. For instance, in newborns exposed to maternal gestational diabetes mellitus (GDM) in utero compared to control subjects, only two DMRs were identified without significant DMPs [38]. Therefore, we evaluated methylation differences not only on the individual CpG level but also the regional level using a dimension reduction approach (comb-p). Our analysis revealed 2023 DMRs in current smokers and 76 DMRs in former smokers in the context of 5mC+5hmC. The DMRs associated with smoking exhibited a substantial overlap with the DMP results in both current and former smokers. Notably, CpG sites within these regions were annotated to previously reported genes, including *GFI1*. In addition, a few annotated genes were exclusively identified in the DMRs results; some examples include *RARRES2*, *RNF40* and *SLC1A5*, associated with current smoking, and *PRRT1*, linked to former smoking. Our findings highlight the importance of regional analysis as an additional approach to validate known or identify novel smoking-related genes. Cigarette smoking is linked to increased cancer incidence and poorer cancer-related clinical outcomes. The results of the enrichment analyses also suggest that the discerned smoking-related effects on DNA methylation are likely to carry implications for the risk of various pathologies, including cardiovascular diseases and cancers.

In the present study, oxBS conversion allowed the specific measurement of nucleotide-level 5mC, which holds promise as a biomarker for various diseases [39] and accurate measurement of the true 5mC signal is crucial to prevent false positive findings. In our study, all significant 5mC DMPs associated with current smoking were also found in the conventional 5mC+5hmC dataset, such as *AHRR*, *RARA* and *F2RL3*, proving that these CpG sites are strongly related to smoking. Furthermore, we noted a substantial concordance in the direction of effects between 5mC+5hmC and 5mC groups in current smokers, with a majority of loci displaying hypomethylation. For example, *AHRR* hypomethylation, serving as an epigenetic marker of smoking history, was reported to predict the risk of myocardial infarction, particularly in former smokers [33]. The CpG site cg24476099, annotated to *MLK1*, emerged as the sole novel significant 5mC linked to former smoking in this study. It is noteworthy that prior research has identified other CpG sites annotated to *MLK1*, demonstrating associations with smoking, incident COPD and prevalent type 2 diabetes [40].

Different methylation modifications possess distinct properties, including varying affinities to transcription factors. Unlike 5mC, often linked to gene repression, 5hmC can inhibit the binding to transcriptional repressors and thereby display the repressive impact of 5mC [41,42]. Hence, the differentiation between 5mC and 5hmC is essential to comprehending the underlying molecular alterations associated with smoking. Most tissues contain approximately 4% 5mC, whereas 5hmC content varies and is typically below 1% in various tissue types [43]. The abundance of 5hmC is remarkably higher in adult neurons and during embryogenesis [44]. Previous research has identified 67 5hmC DMPs between healthy smokers and non-smokers using lung bronchoalveolar lavage cells, providing evidence of 5hmC being involved in the effects of smoking. These findings also suggested that smoking-related differences may involve DNA demethylation of 5mC with a 5hmC intermediate, as inferred from the observed contrasting hypomethylated 5mC and hypermethylated 5hmC data [45]. Our study aligns with this interpretation, further supporting the notion that smoking-induced oxidative stress can trigger DNA demethylation through the sequential oxidation procedure. As expected, given its low abundance in blood, the DNA hydroxymethylation signature linked to smoke exposure exhibited a lesser prominence compared to true DNA methylation, even under a less stringent threshold. The CpG sites cg16972043 (annotated to *GPT2*) and cg24012880 (annotated to *TSPAN18*) emerged as the most significant and novel hydroxymethylated CpG sites associated with current and former smoking, respectively. *GPT2* serves as a crucial link between glycolysis and glutaminases and exhibits significant upregulation in aggressive breast cancers [46]. Recent research has unveiled *GPT2*’s role in regulating smoking-induced metabolism and damage in airway epithelial cells through its impact on lipid synthesis [47]. Furthermore, both *GPT2* and *TSPAN18* have been implicated in incident COPD in leukocytes [40], underscoring their relevance in respiratory conditions. The identification of these novel smoking-associated hydroxymethylated CpG sites holds promise for guiding future research endeavours. The present study has several strengths. Our multivariate linear regression model was meticulously adjusted for many potential confounders, including estimated cell fractions. To enhance the precision of our findings, we differentiated between true 5mC and 5hmC signals using the tandem BS and oxBS treatment, effectively minimizing the likelihood of identifying false positives, especially in combination with Infinium Methylation EPIC BeadChip. Additionally, the study’s robustness was further fortified by the assessment of DMRs in addition to individual CpG sites. However, our study does have limitations. Passive smoking was not considered, and additional continuous smoking variables like pack years were unavailable, limiting the comprehensive analysis of smoking effects. The absence of a replication cohort emphasizes the need for future studies to validate our findings in independent populations. Additionally, the use of DNA derived from blood may not fully capture tissue-specific variations in methylation patterns; exploring specific tissues could offer more nuanced information on the impact of smoking on both true DNA methylation and hydroxymethylation.

## 5. Conclusions

Our results confirmed previously reported smoking-associated CpG sites with the Illumina Infinium Methylation EPIC BeadChip, but also revealed many novel smoking-associated signatures. By distinguishing 5mC and 5hmC data from peripheral blood DNA samples, our study identified distinct smoking-associated DNA methylation modifications. Hydroxymethylation was not strongly associated with smoking in peripheral blood DNA samples, but suggestive hydroxymethylated CpG sites might inform future research.

## Figures and Tables

**Table 1:** Characteristics of the study population.

Characteristics	All Participants	Current Smokers	Former Smokers	Non-Smokers
	1717	217	719	781
Age (years)	63 (59, 68)	61 (57, 65) ***	64 (59, 68)	63 (59, 68)
Male (%)	814 (46.3%)	105 (47.3%)	393 (53.5%) ^###^	316 (39.4%)
BMI (kg/m^2^)	27.4 (24.5, 30.8)	26.2 (23.7, 30)	27.6 (24.8, 31.3) ^#^	27.3 (24.5, 30.3)
Physical activity	1268 (72.1%)	159 (71.6%)	535 (72.8%)	574 (71.6%)
Alcohol intake (g/day)	6.6 (0, 22.9)	8.6 (0, 30) *	8.6 (0.2, 23.8) ^##^	5.7 (0, 20)
Hypertension	855 (48.7%)	82 (36.9%) *	395 (53.8%) ^#^	378 (47.2%)
Diabetes mellitus	135 (7.7%)	14 (6.3%)	65 (8.9%)	56 (7%)
HDL-cholesterol (mg/dL)	61.7 (51.1, 75)	58.5 (49, 69.9) ***	61.2 (50, 75) ^#^	62.8 (53, 77.2)
LDL-cholesterol (mg/dL)	122.8 (99.1, 146.5)	124.7 (99.9, 147.4)	119.6 (95.6, 144) ^##^	126.2 (103, 147.8)
Total cholesterol (mg/dL)	212.4 (185.1, 238.3)	211.9 (184.4, 234.7)	208.9 (181.8, 236.1) ^##^	215.8 (189.6, 241.9)
Triglycerides (mg/dL)	106 (77.7, 145.6)	109.3 (85.4, 153.5) *	107.7 (77.9, 149.2) ^#^	103 (76.2, 139)
Fasting glucose (mg/dL)	98 (92, 107)	96 (91, 104)	100 (93, 109) ^###^	97 (92, 105)
HOMA-IR	2.3 (1.5, 3.5)	2.1 (1.4, 3)	2.3 (1.5, 3.6)	2.3 (1.5, 3.4)
HOMA-Beta	97.8 (71.2, 132)	93.1 (68.7, 124.2)	97.1 (68.9, 132.3)	101 (73.9, 132.7)
HbA1c (%)	5.5 (5.3, 5.8)	5.6 (5.3, 5.8) *	5.5 (5.3, 5.8)	5.5 (5.2, 5.8)

Basic characterization of individuals in our cohort. Continuous variables are presented as median (25th, 75th), while categorical variables are expressed as *n* (%). Statistical analyses employed the Kruskal–Wallis Test for continuous variables and the Chi-square test for categorical variables. Significance levels for comparisons between current and non-smokers are denoted as * *p* < 0.05, *** *p* < 0.001. For comparisons between former and non-smokers, significance levels are indicated as ^#^ *p* < 0.05, ^##^ *p* < 0.01, ^###^ *p* < 0.001.

**Table 2:** Summary of top 10 most significant 5mC+5hmC DMPs from current and former smokers.

Probe	Delta Beta	*p* Value	FDR	CHR	Gene	MAPINFO	EPIC
**Current DMPs**	data	data					
cg05575921	−22.72%	2.13 × 10^−245^	1.56 × 10^−239^	5	*AHRR*	373378	
cg21566642	−16.26%	1.89 × 10^−162^	6.94 × 10^−157^	2		233284661	
cg01940273	−9.67%	5.22 × 10^−147^	1.27 × 10^−141^	2		233284934	
cg03636183	−9.88%	5.45 × 10^−140^	1.00 × 10^−134^	19	*F2RL3*	17000585	
cg21161138	−6.88%	1.91 × 10^−111^	2.80 × 10^−106^	5	*AHRR*	399360	
cg17739917	−10.21%	4.62 × 10^−110^	5.65 × 10^−105^	17	*RARA*	38477572	*
cg14391737	−10.12%	5.50 × 10^−82^	5.77 × 10^−77^	11	*PRSS23*	86513429	*
cg26703534	−4.88%	1.90 × 10^−78^	1.75 × 10^−73^	5	*AHRR*	377358	
cg17087741	−6.13%	4.22 × 10^−77^	3.44 × 10^−72^	2		233283010	
cg21911711	−5.65%	1.44 × 10^−71^	1.06 × 10^−66^	19	*F2RL3*	16998668	*
**Former DMPs**							
cg14391737	−4.56%	2.23 × 10^−40^	1.63 × 10^−34^	11	*PRSS23*	86513429	*
cg21566642	−4.62%	1.74 × 10^−36^	6.40 × 10^−31^	2		233284661	
cg05575921	−4.06%	1.20 × 10^−25^	2.95 × 10^−20^	5	*AHRR*	373378	
cg06644428	−2.20%	3.45 × 10^−23^	6.34 × 10^−18^	2		233284112	
cg01940273	−2.24%	1.74 × 10^−22^	2.56 × 10^−17^	2		233284934	
cg16841366	−2.62%	2.90 × 10^−16^	3.56 × 10^−11^	2		233286192	*
cg11660018	−1.65%	4.39 × 10^−16^	4.61 × 10^−11^	11	*PRSS23*	86510915	
cg00475490	−1.53%	1.04 × 10^−15^	9.56 × 10^−11^	11	*PRSS23*	86517110	*
cg03636183	−1.88%	5.66 × 10^−15^	1.35 × 10^−9^	19	*F2RL3*	17000585	
cg17739917	−2.20%	1.85 × 10^−14^	1.35 × 10^−9^	17	*RARA*	38477572	*
cg14391737	−4.56%	2.23 × 10^−40^	1.63 × 10^−34^	11	*PRSS23*	86513429	*

Probe: Unique identifier from the Illumina CG database; Delta Beta: Mean methylation difference between smokers and non-smokers; FDR: Benjamini–Hochberg corrected *p* value (FDR); CHR: Chromosome; Gene: Target gene name from the UCSC database; MAPINFO: Chromosomal coordinates of the CpG (Build 37); EPIC: * indicates CpG sites that are exclusively present in the Infinium Methylation EPIC BeadChip.

**Table 3:** Summary of significant true 5mC and 5hmC DMPs from current and former smokers.

Probe	Delta Beta	*p* Value	FDR	CHR	Gene	MAPINFO	EPIC
**5mC Current**	data	data					
cg05575921	−24.01%	1.68 × 10^−77^	1.27 × 10^−71^	5	*AHRR*	373378	
cg21566642	−14.63%	2.26 × 10^−34^	8.58 × 10^−29^	2		233284661	
cg01940273	−9.32%	2.02 × 10^−26^	5.10 × 10^−21^	2		233284934	
cg03636183	−8.41%	7.61 × 10^−25^	1.43 × 10^−19^	19	*F2RL3*	17000585	
cg14391737	−11.13%	6.90 × 10^−17^	1.04 × 10^−11^	11	*PRSS23*	86513429	
**5mC Former**							
cg24476099	−4.34%	3.95 × 10^−8^	0.03	22	*MKL1*	40925033	*
**5hmC Current**	−4.62%	1.74 × 10^−36^	6.40 × 10^−31^	2		233284661	
cg16972043	4.14%	1.36 × 10^−7^	0.103	16	*GPT2*	46932066	*
cg01483713	1.97%	1.89 × 10^−6^	0.718	4		6252582	*
cg15297506	1.22%	4.42 × 10^−6^	0.784	10	*SH3PXD2A*	105453418	*
cg04131101	3.50%	4.90 × 10^−6^	0.784	11		94427846	
cg22377040	1.68%	5.40 × 10^−6^	0.784	6	*TRIM31*	30071412	
**5hmC Former**	−1.53%	1.04 × 10^−15^	9.56 × 10^−11^	11	*PRSS23*	86517110	*
cg24012880	3.61%	4.45 × 10^−7^	0.337	11	*TSPAN18*	44880910	
cg10148425	2.58%	6.77 × 10^−6^	0.985	19		184224630	*

Probe: Unique identifier from the Illumina CG database; Delta Beta: Mean methylation difference between smokers and non-smokers; FDR: Benjamini–Hochberg corrected *p* value (FDR); CHR: Chromosome; Gene: Target gene name from the UCSC database; MAPINFO: Chromosomal coordinates of the CpG (Build 37); EPIC: * indicates CpG sites that are exclusively present in the Infinium Methylation EPIC BeadChip.

**Table 4:** Summary of top 10 most significant total 5mC+5hmC DMRs from current and former smokers.

Gene	CHR	Start	End	*p* Value	FDR	Nprobe
**Current smokers**						
	2	233283010	233286291	5.02 × 10^−212^	3.97 × 10^−208^	12
*GFI1*	1	92945668	92947962	5.74 × 10^−130^	3.03 × 10^−126^	9
*AHRR*	5	399360	400833	1.16 × 10^−63^	2.29 × 10^−60^	7
*C5orf62*	5	150161299	150162069	7.24 × 10^−53^	8.20 × 10^−50^	3
*SLC1A5*	19	47287778	47289612	3.52 × 10^−51^	3.72 × 10^−48^	12
	19	1265877	1266000	1.66 × 10^−48^	1.65 × 10^−45^	3
	14	106329158	106331863	2.67 × 10^−46^	2.49 × 10^−43^	19
*HIVEP3*	1	42384002	42385942	5.62 × 10^−46^	4.69 × 10^−43^	15
*ITGAL*	16	30485296	30485967	1.09 × 10^−44^	8.68 × 10^−42^	7
	6	30719807	30720485	4.34 × 10^−42^	2.86 × 10^−39^	6
**Former smokers**						
	2	233283010	233286291	1.53 × 10^−61^	2.38 × 10^−59^	12
*PRRT1*	6	32118204	32118458	4.68 × 10^−22^	1.81 × 10^−20^	13
*NBL1*	1	19971709	19972778	2.37 × 10^−17^	7.37 × 10^−16^	9
	19	1265877	1266000	2.98 × 10^−16^	7.71 × 10^−15^	3
*ANPEP*	15	90345999	90346095	8.64 × 10^−16^	1.91 × 10^−14^	3
	1	161708999	161710014	2.05 × 10^−13^	3.17 × 10^−12^	3
*PRSS23*	11	86510915	86511218	8.38 × 10^−13^	1.18 × 10^−11^	5
*PPT2*	6	32120955	32121556	1.70 × 10^−12^	2.19 × 10^−11^	20
*VARS*	6	31762353	31762902	3.91 × 10^−12^	3.56 × 10^−11^	15
*GNA12*	7	2847477	2847576	1.47 × 10^−11^	1.26 × 10^−10^	3
	2	233283010	233286291	1.53 × 10^−61^	2.38 × 10^−59^	12

Gene: UCSC gene name; CHR: Chromosome; Start: Start CHR position of this region; End: End CHR position of this region; FDR: Benjamini–Hochberg corrected *p* value; Nprobe: number of CpG probes in this region.

## Data Availability

Data are contained within the article and Supplementary Files. The KORA data are available upon request from the KORA Project Application Self-Service Tool (https://www.helmholtz-munich.de/en/epi/cohort/kora, accessed on 10 April 2024).

## Supplementary Materials

The following supporting information can be downloaded at: https://www.mdpi.com/article/10.3390/biom14060662/s1, Figure S1: QQ plots for total 5mC+5hmC methylation; Figure S2: Volcano plots of smoking association effect sizes for total 5mC+5hmC methylation; Figure S3: Manhattan plots of DMR results for total 5mC+5hmC methylation, Figure S4: Volcano plots of smoking association effect sizes for 5mC and 5hmC methylation, Figure S5: QQ plots for 5mC and 5hmC methylation; Figure S6: Manhattan plots of DMR results for 5mC methylation; Figure S7: Gene enrichment analysis plots of true 5mC and 5hmC methylation. Tables S1–S2: the significant DMPs related to current and former smoking from total 5mC+5hmC methylation dataset; Tables S3–S4: the novel DMPs related to current and former smoking from total 5mC+5hmC methylation dataset; Tables S5–S6: the significant DMPs related to current and former smoking from 5mC methylation dataset; Tables S7–S8: the significant DMPs related to current and former smoking from 5hmC methylation dataset. Tables S9–S12: the significant DMRs related to current and former smoking from total 5mC+5hmC and true 5mC methylation datasets; Tables S13–S18: the pathways related to current and former smoking from total 5mC+5hmC, true 5mC and 5hmC methylation datasets.
