# Supplementary material for: Smoking-Induced DNA Hydroxymethylation Signature Is Less Pronounced than True DNA Methylation: The Population-Based KORA Fit Cohort
Source: Biomolecules. 2024 Jun 5;14(6):662. doi: 10.3390/biom14060662 (PMC11201877; doi:10.3390/biom14060662)
Supplement: Supplementary file 1 [file biomolecules-14-00662-s001.zip › Supplementary Material 1.pdf]

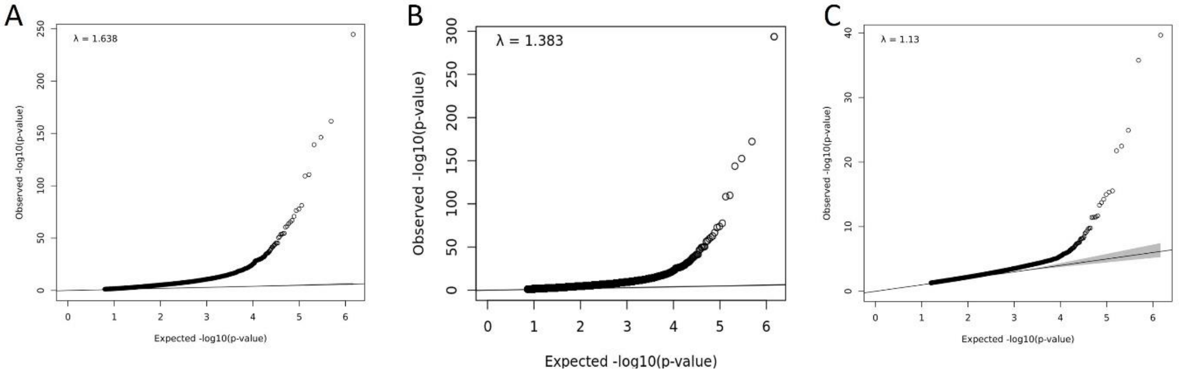

Figure S1. QQ plots for total 5mC+5hmC methylation. The x-axis represents the expected –log10 (P-value) and the y-axis represents the observed –log10 (P-value). **(A)** QQ plot for current vs non-smokers; **(B)** QQ plot for current vs non-smokers after bacon correction; **(C)** QQ plot for former vs non-smokers.

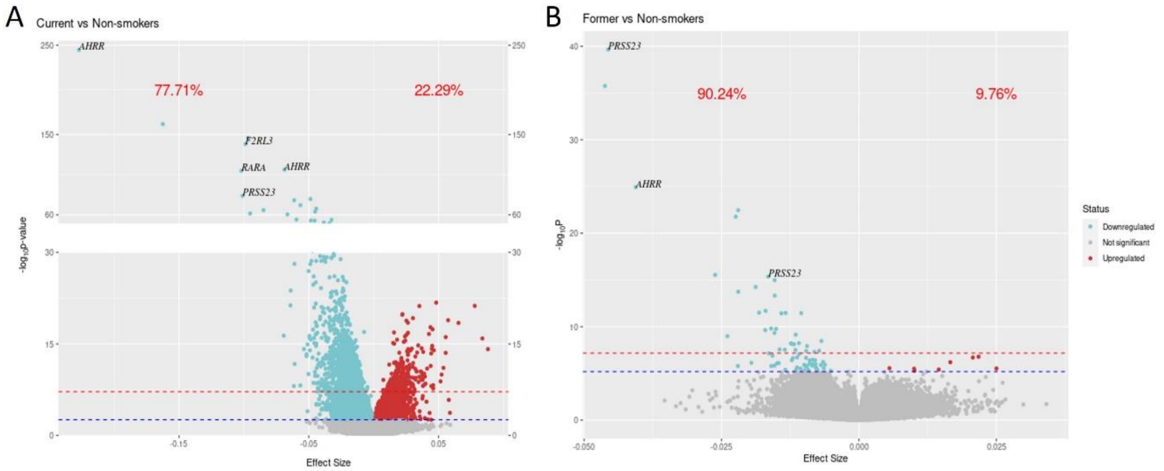

Figure S2. Volcano plots of smoking association effect sizes for total 5mC+5hmC methylation. The x-axis represents the effect size (the methylation value difference between groups), and the y-axis represents the –log10 (*P*-value). The Bonferroni threshold of 6.81×10^{-8} is marked by a red dashed line, while the Benjamini-Hochberg (FDR) threshold (*P*<0.05) is indicated by a blue dashed line. The ggbreak package was used to effectively utilize plotting space and handle large y-axis for currents smokers. **(A)** Volcano plot for current vs non-smokers; **(B)** Volcano plot for former vs non-smokers.

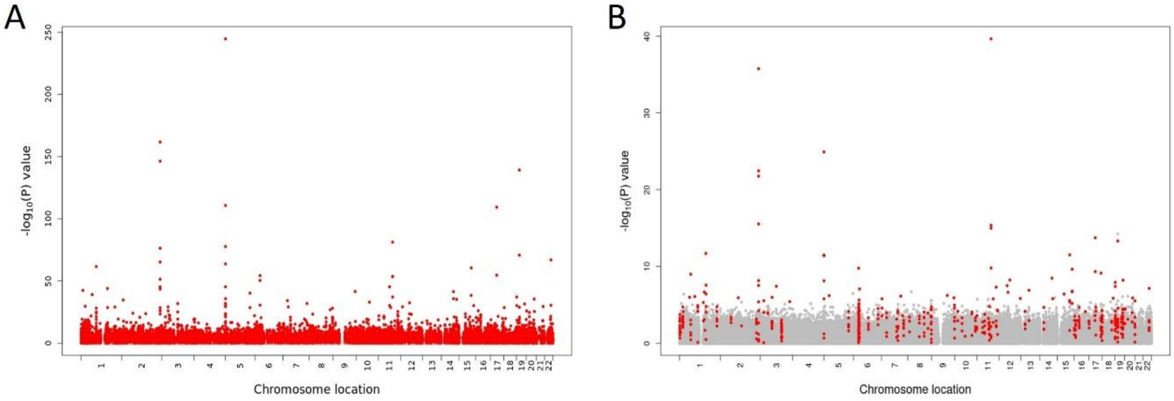

Figure S3. Manhattan plots of DMR results for total 5mC+5hmC methylation. The x-axis represents the chromosome location, and the y-axis represents the –log10 (*P*-value). **(A)** Manhattan plot for current vs non-smokers; **(B)** Manhattan plot for former vs non-smokers.

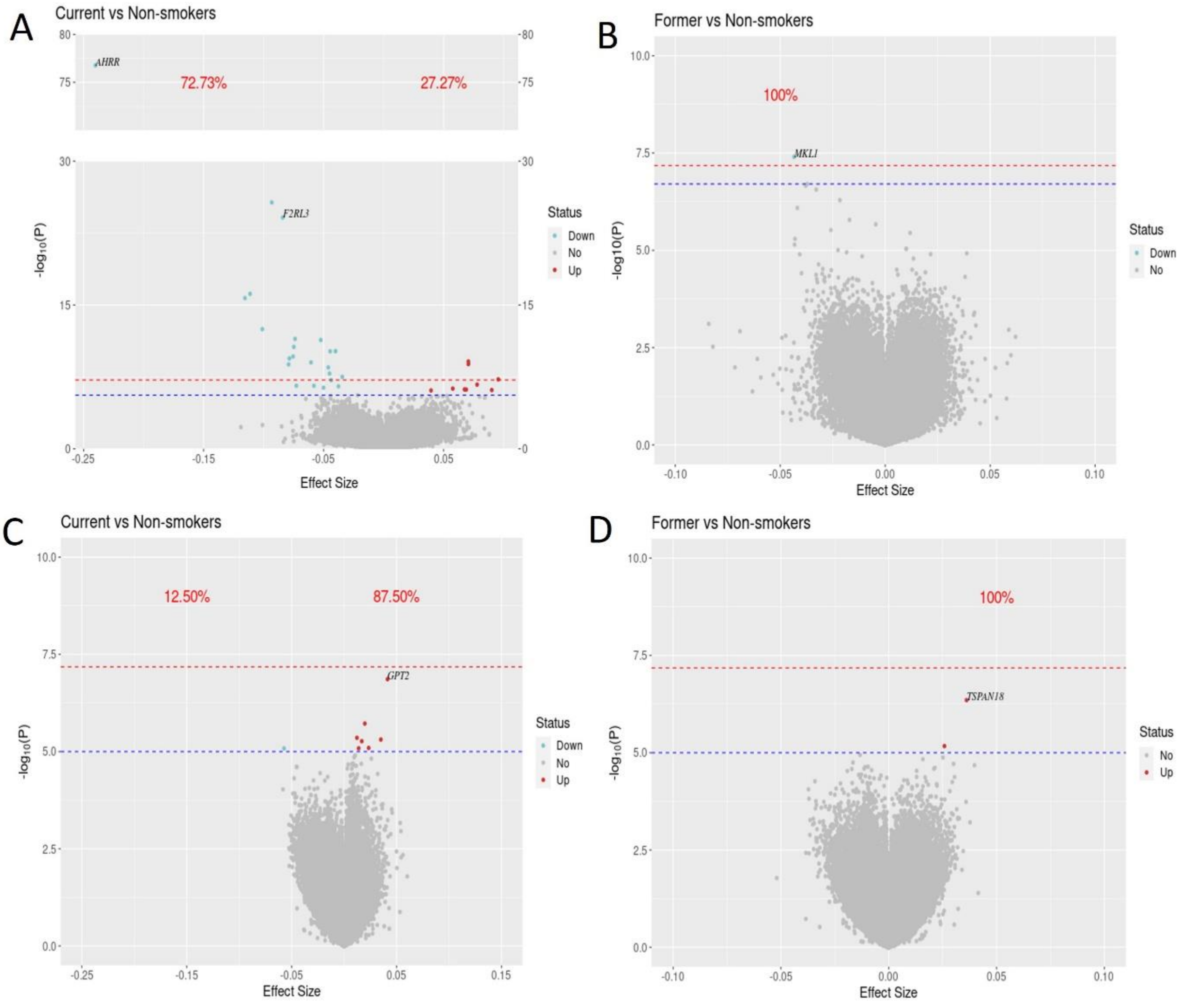

Figure S4. Volcano plots of smoking association effect sizes for 5mC and 5hmC methylation. The x-axis represents the effect size (the methylation value difference between groups), and the y-axis represents the –log10 (*P*-value). The Bonferroni threshold of 6.61×10^{-8} is marked by a red dashed line, while the Benjamini-Hochberg (FDR) threshold ($P<0.05$) is indicated by a blue dashed line. The ggbreak package was used to effectively utilize plotting space and handle large y-axis for

currents smokers. **(A)** Volcano plot for current vs non-smokers from 5mC dataset; **(B)** Volcano plot for former vs non-smokers from 5mC dataset; **(C)** Volcano plot for current vs non-smokers from 5hmC dataset; **(D)** Volcano plot for former vs non-smokers from 5hmC dataset.

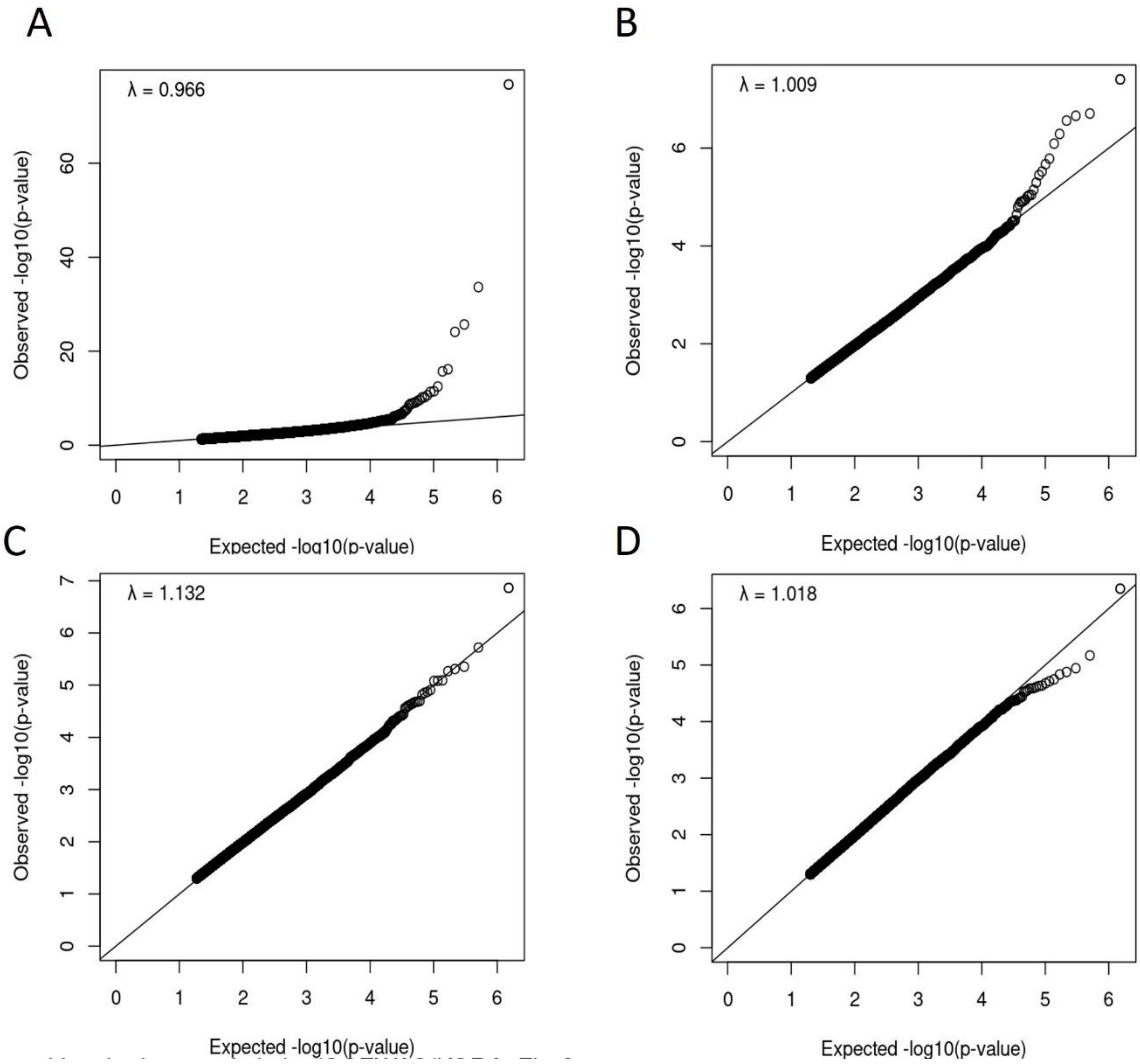

Figure S5. QQs plots of for 5mC and 5hmC methylation. The x-axis represents the expected –log10 (P-value) and the y-axis represents the observed –log10 (*P*-value). **(A)** QQ plot for current vs non-smokers from 5mC dataset; **(B)** QQ plot for former vs non-smokers from 5mC dataset; **(C)** QQ plot for current vs non-smokers from 5hmC dataset; **(D)** QQ plot for former vs non-smokers from 5hmC dataset.

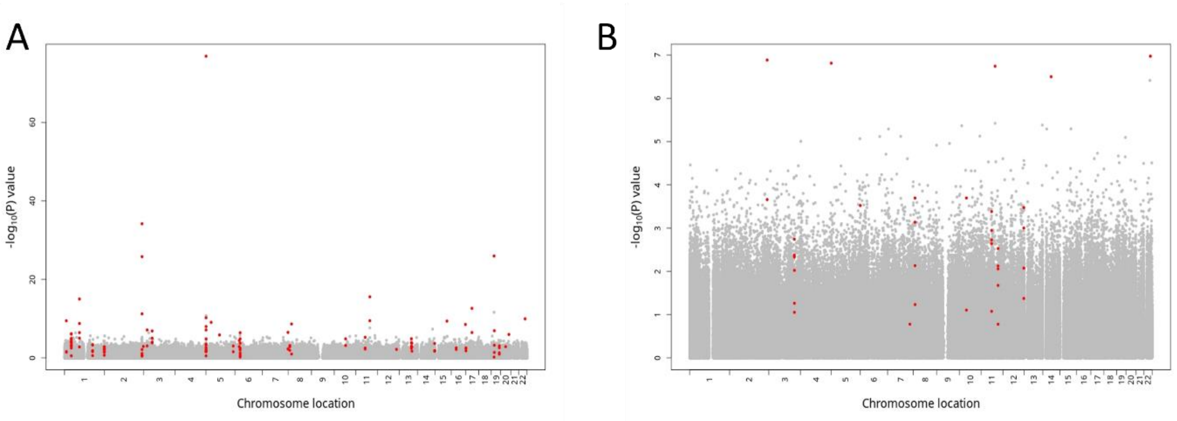

Figure S6. Manhattan plots of DMR results for 5mC methylation. The x-axis represents the chromosome location, and the y-axis represents the –log10 (*P* value). **(A)** Manhattan plot for current vs non-smokers from 5mC dataset; **(B)** Manhattan plot for former vs non-smokers from 5mC dataset.

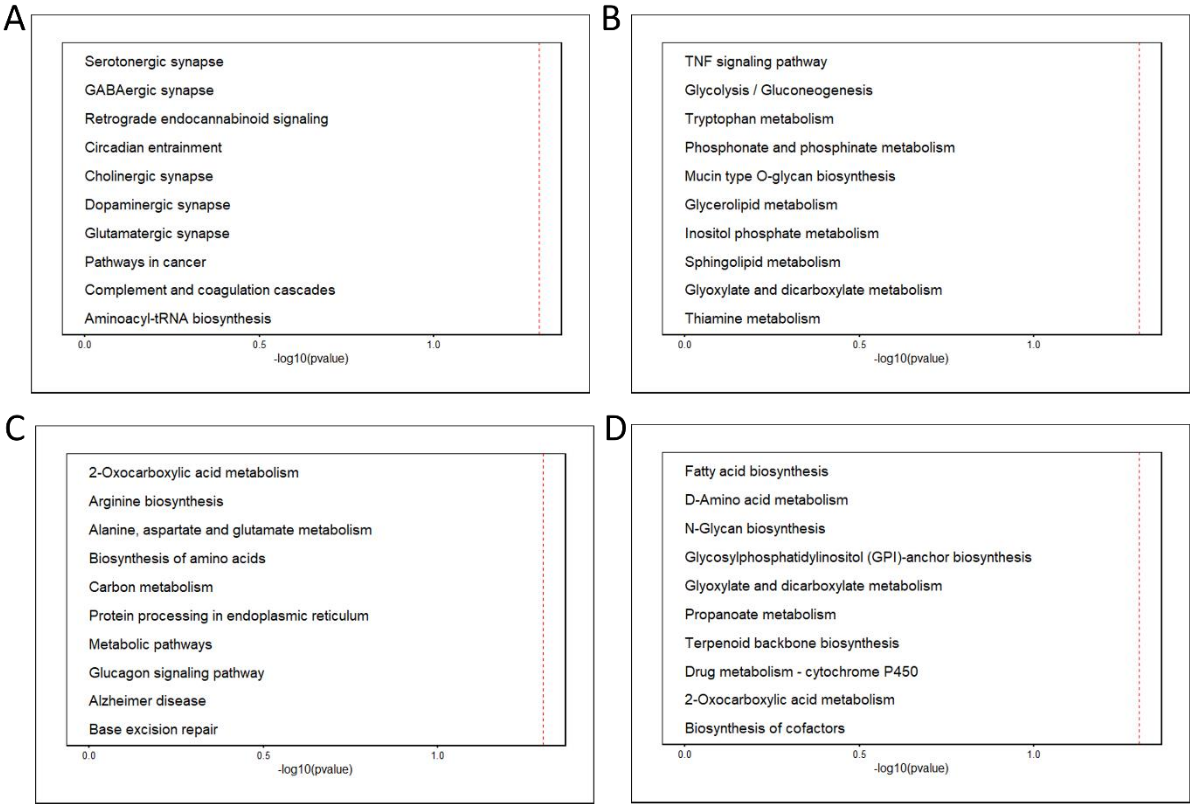

Figure S7. Gene enrichment analysis plots of true 5mC and 5hmC methylation. The x axis represents the -log10(*P*-value), and the red dashed line represents the significant threshold (FDR-adjusted $P<0.05$). (A) illustrate the top 10 pathways derived from true 5mC methylation between current vs non-smokers. (B) illustrate the top 10 pathways derived from true 5mC methylation between former vs non-smokers. (C) illustrate the top 10 pathways derived from 5hmC methylation between current vs non-smokers. (D) illustrate the top 10 pathways derived from 5hmC methylation between former vs non-smokers.
